# MicroRNA-451 regulates stemness of side population cells via PI3K/Akt/mTOR signaling pathway in multiple myeloma

**DOI:** 10.18632/oncotarget.3802

**Published:** 2015-04-12

**Authors:** Juan Du, Shuyan Liu, Jie He, Xi Liu, Ying Qu, Wenqing Yan, Jianling Fan, Rong Li, Hao Xi, Weijun Fu, Chunyang Zhang, Jing Yang, Jian Hou

**Affiliations:** ^1^ Department of Hematology, The Myeloma and Lymphoma Center, Changzheng Hospital, The Second Military Medical University, Shanghai, China; ^2^ Department of Lymphoma/Myeloma, Division of Cancer Medicine and Center for Cancer Immunology Research, The University of Texas, MD Anderson Cancer Center, Houston, Texas, USA

**Keywords:** multiple myeloma, side population, miRNA-451, PI3K/Akt/mTOR, stemness

## Abstract

Side population (SP) cells are an enriched source of cancer-initiating cells with stemness characteristics, generated by increased ABC transporter activity, which has served as a unique hallmark for multiple myeloma (MM) stem cell studies. Here we isolated and identified MM SP cells via Hoechst 33342 staining. Furthermore, we demonstrate that SP cells possess abnormal cell cycle, clonogenicity, and high drug efflux characteristics-all of which are features commonly seen in stem cells. Interestingly, we found that bortezomib, As_2_O_3_, and melphalan all affected apoptosis and clonogenicity in SP cells. We followed by characterizing the miRNA signature of MM SP cells and validated the specific miR-451 target tuberous sclerosis 1 (TSC1) gene to reveal that it activates the PI3K/Akt/mTOR signaling in MM SP cells. Inhibition of miR-451 enhanced anti-myeloma novel agents' effectiveness, through increasing cells apoptosis, decreasing clonogenicity, and reducing MDR1 mRNA expression. Moreover, the novel specific PI3K/Akt/mTOR signaling inhibitor S14161 displayed its prowess as a potential therapeutic agent by targeting MM SP cells. Our findings offer insights into the mechanisms regulating MM SP cells and provide a novel strategy to overcome resistance to existing therapies against myeloma.

## INTRODUCTION

Multiple myeloma (MM) is a clonal B-cell malignancy with terminally differentiated plasma cells. Despite remarkable progress made in the biology and treatment of the disease, it is still incurable [1, 2]. It has long been postulated that a small population of cancer stem cells (CSCs) persist in bone marrow niches resulting in the development of refractory clones and disease relapse [3].

Previous groups have successively described various CSC phenotypes in MM, including CD138-/CD34- with memory B-cells (CD19+/CD27+) [4, 5], CD138-/CD19+ cells with cytoplasmic light chain-restricted (LCR) cells [6], CD19-/CD45low-/CD38high/CD138+ [7], and CD138+ cell [8]. However, the distinct myeloma CSC marker is still one of the most controversial issues. Side population (SP) cells, first described by Goodell et al.[9], are a subset of enriched progenitor cells exhibiting stem-like phenotypes with a distinct low Hoechst 33342, which has been widely used as a unique source for studying CSC when specific markers are unavailable. Even though a few studies have previously explored the stem-like properties and tumorigenicity of SP cells in comparison to the main population (MP) cells, an understanding of MM SP cells remain elusive [5, 10-13].

MicroRNAs (miRNAs) are a class of regulatory non-coding RNAs approximately 22 nucleotides in length and function primarily by targeting specific mRNA sequences [14]. MiRNAs have emerged as important regulators of CSCs, which lead to the initiation, development, and progression of many cancers [15, 16]. The signaling pathway components regulating normal stem cell self-renewal are commonly aberrantly activated in many human cancers, serving as potential therapeutic targets. The signaling pathways are ideal candidates for miRNA-mediated regulation due to the sharp dose-sensitive nature of regulatory effects [17]. In the case of MM SP cells, however, miRNAs serving as nodes of the cell signaling pathways are yet defined. Therefore, the goal of this study was to identify and investigate stem-like cell properties of MM SP cells through modulating miRNAs involved in the relevant signaling pathways so to advance development of novel and effective MM therapies targeting SP cells.

## RESULTS

### SP cells existed in MM cell lines and primary MM cells

To isolate and provide evidence that the SP cells exist in myeloma cells, we analyzed a panel of cell lines, including NCI-H929, RPMI 8226, KMS-11, LP-1, U266, and SKO, as well as seven primary myeloma cells with Hoechst 33342 staining. Cells were also incubated in the presence of verapamil (100 μM), a chemical inhibitor of the ABC protein family of transporters, which inhibits the efflux of Hoechst as the negative control for SP cell. The MM cell lines were found to contain of SP cells, presented as a distinct “tail” in the flow cytometry analysis (Supplemental Fig. 1A and 1B), from 0.5%~10.3% and patients' samples ranged a minor population of SP from 0.04%~2.0% (Supplemental Fig. 1C and 1D). We then utilized NCI-H929 and KMS-11 cell lines, both of which showed a reliable and stable detected SP cell population, to further investigate the functional characteristics of SP cells.

### SP cells endowed with distinct stem cell characteristics in MM

**Figure 1:**
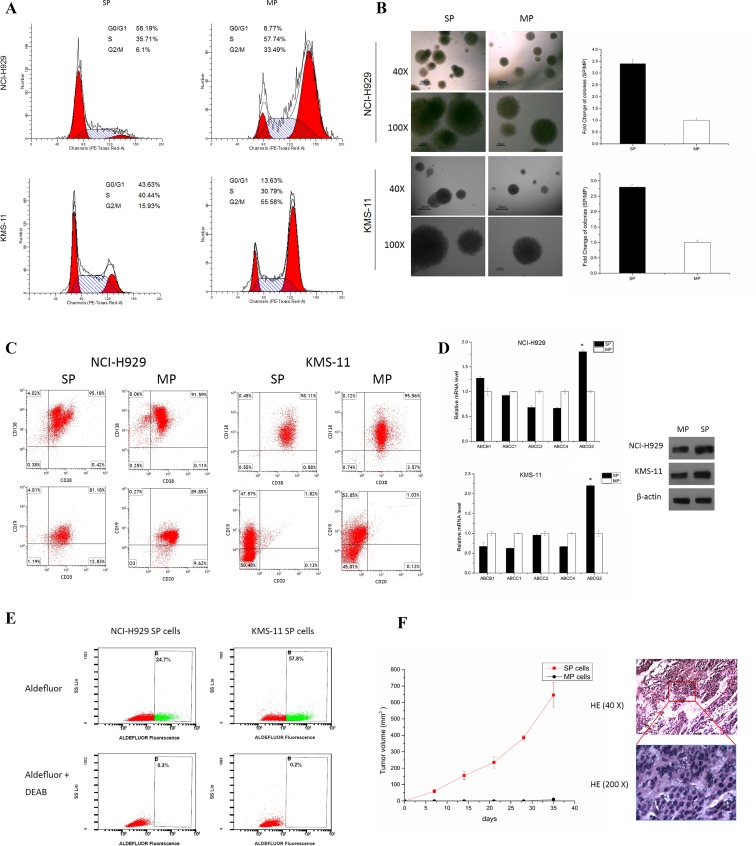
SP cells possess stem cell properties **A**. Cell cycle analysis of SP or MP cells isolated from the NCI-H929 and KMS-11 cell lines after PI staining. **B**. CFC assay in sorted SP and MP cells isolated from the NCI-H929 and KMS-11cell lines was assessed after 14 days of culture (200 cells/plate). Representative images of colonies of sorted SP and MP are shown using an inverted microscope with a Nikon DXM1200F camera (magnification 40 × & 100 ×). **C**. Cell surface phenotype of SP cells by fluorescence immunophenotyping assay for CD38, CD138, CD19, and CD20 expression in NCI-H929 and KMS-11 cells. **D**. qRT-PCR showing relative ABC transporter mRNA levels in SP and MP cells sorted from NCI-H929 and KMS-11 cells, respectively. ABCG2 expression increases significantly under stem cell conditions by qRT-PCR and western blot assays (*p < 0.05). **E**. ALDH activity of SP and MP cells sorted from NCI-H929 and KMS-11 cells. A specific ALDH inhibitor (DEAB) as a negative control. Graph shows percentages of cells with increased ALDH activity within SP populations. All experiments were performed in triplicates. **F**. Tumorigenic formation potential of SP and MP cells. NOD/SCID mice were subcutaneously inoculated with SP or MP cells from 1×10^5^ cells/mice. Caliper measurements of tumor diameters were measured every 7 days. H&E stained images of tumor from SP mice groups with magnification of the selected areas. Scale bar, 40×, 200 μm. 200×, 50 μm.

SP cells have inherent properties that distinguish them from MP cells from their less-differentiated progeny. To determine if MM stem-like cells exist in a relatively quiescent state, SP and MP cells isolated from the NCI-H929 and KMS-11 cell lines were examined for their cell cycle status. We found that NCI-H929 SP cells contained a significantly higher percentage cells in G1/G0 phase (58.19%) compared to MP cells, which only contained 8.77% of cells in G1/G0 phase (Fig. 1A). Approximately 35.71% and 6.1% of SP cells were in S and G2 phases, respectively, compared to 57.74% and 33.49% in MP cells. Similarly, a high percentage cells in G1/G0 phase were detected in SP cells in comparison to MP cells of KMS-11 cell line (Fig. 1A).

We next examined the self-renewal capacity of SP cells. We found SP cells to have high clonegenicity, requiring only 200 cells/plate to successfully grow into colonies (Fig. 1B). After 14 days in culture, SP cells generated larger and around 3 times as many the number of colonies derived from MP cells either in NCI-H929 or KMS-11 cell lines, demonstrating that SP cells have a stronger self-renewal and proliferative abilities.

In MM, the immunophenotyping of CSC is still a controversial topic [4, 5, 12, 13]. We stained the cells with CD38, CD138, CD20, and CD19 antibodies to characterize the immunophenotypes of NCI-H929 and KMS-11. Consistent with previous studies [13], we observed both CD138- and CD138+ expression on SP and MP cells, with no significant difference in expression level between the two populations. We found no significant difference in CD20 and CD19 expression between SP and MP cells (Fig. 1C).

ABCB1 (MDR1), ABCC1 (MRP1), ABCC2 (MRP2), ABCC4 (MRP4), and ABCG2 (BRCP) are the main transporters in multidrug resistance (MDR) family known to possess the ability to exclude drugs as well as being present in CSCs. We detected expression of these transporters in SP and MP sorted from NCI-H929 and KMS-11 cells. ABCG2 transcripts were significantly enhanced in SP cells in comparison to MP cells in both myeloma cell lines. However, the expressions of ABCB1 was slight enhanced in SP cells compared to MP sorted from NCI-H929 cells, while ABCC1, ABCC2, and ABCC4 were somewhat reduced in both cell lines (Fig. 1D). And then, the ABCG2 protein expression level were confirmed by western blot assay (Fig. 1D). This observation is consistent with the concept of ABCG2 transporter being the most specific SP marker from the MDR family [18].

Recently, detection of ALDH activity has been touted as a marker of hematopoietic stem/progenitor cells [19]. To determine whether SP cells contain the high ALDH expression as well, we used the fluorescent Aldefluor assay to exam ALDH activity. We observed a significantly increase in Aldefluor activity in NCI-H929 SP cells (57.8 %) and KMS-11 SP cells (24.7%) than compared to corresponding MP cells (Fig. 1E).

To evaluate tumorigenicity of SP cells in mice, a total of 1×10^5^ of SP or MP cells was injected subcutaneously into NOD/SCID mice with 6 mice per group per time points. We observed two out of six mice injected with SP cells developed subcutaneous tumors with great tumor mass, whereas mice injected with MP cells did not develop any tumor (Fig. 1F). These results, combined with our previous studies, showed only a 40% tumor formation rate in NCI-H929 cells. We thus speculated that the tumorigenicity of NCI-H929 derives from SP cells since only SP fractions eventually developed tumors with the latency and tumor mass being consistent with previously reported data [8, 13].

**Table 1:** Differential expression of miRNA identified in SP and MP cells

miRNA	Chromosome location	Fold change		*p*-value
**Upregulated miRNA**				
hsa-miR-33b	17	6.987367677		0.0341726533
hsa-miR-326	11	6.612866664		0.0000050910
hsa-miR-335*	7	4.805920365		0.0207873985
hsa-miR-144	17	4.107045945		0.0016160025
hsa-miR-451	17	3.801646299		0.0030331962
hsa-miR-363	X	3.685533855		0.0147515387
hsa-miR-4290	9	3.408334553		0.0006398131
hsa-miR-486-5p	8	2.741136533		0.0012473680
hsa-miR-3182	16	2.601167475		0.0001489232
hsa-miR-1976	1	2.400666849		0.0000026920
**Downregulated miRNA**				
hsa-miR-155	21	0.442529258		0.0486285773
hsa-miR-30c	1	0.4376255		0.0456339634
hsa-miR-148a	7	0.433821762		0.0433979100
hsa-miR-320d	13	0.423355394		0.0376287918
hsa-miR-193b	16	0.411037164		0.0315374022
hsa-miR-3142	5	0.407958238		0.0301288756
hsa-miR-125a-5p	19	0.407900215		0.0301027596
hsa-miR-548e	10	0.403739023		0.0282706529
hsa-miR-377	14	0.403452896		0.0281476142
hsa-miR-181a	9	0.39171302		0.0234167522
hsa-miR-9	1	0.383712109		0.0205353941
hsa-miR-136	14	0.376078245		0.0180319907
hsa-miR-30e	1	0.356125342		0.0125381209
hsa-miR-320a	8	0.355034273		0.0122785570
hsa-miR-181d	19	0.344328059		0.0099380745
hsa-miR-664*	1	0.334818122		0.0081545737
hsa-miR-335	7	0.327739702		0.0069927283
hsa-miR-127-3p	14	0.324579351		0.0065166949
hsa-miR-153	2	0.315892956		0.0053352003
hsa-miR-30b	8	0.289593177		0.0027367749
hsa-miR-29b	1	0.283806256		0.0023302278
hsa-miR-23b	9	0.279436815		0.0020562097
hsa-miR-29a	7	0.276089317		0.0018641339
hsa-miR-3651	9	0.2701373		0.0015580967
hsa-miR-125b	11	0.25515444		0.0009625090
hsa-miR-150	19	0.245821776		0.0006960970
hsa-miR-129*	7	0.202755244		0.0001156061
hsa-miR-3647-3p	16	0.201213328		0.0001072185
hsa-miR-124	8	0.159268752		0.0000091529
hsa-miR-3607-5p	5	0.156882099		0.0000077280
hsa-miR-3653	22	0.150251473		0.0000047301
hsa-miR-9*	5	0.149422405		0.0000044385
hsa-miR-3607-3p	5	0.078787431		0.0000000009

### SP cells exhibit drug resistance

**Figure 2:**
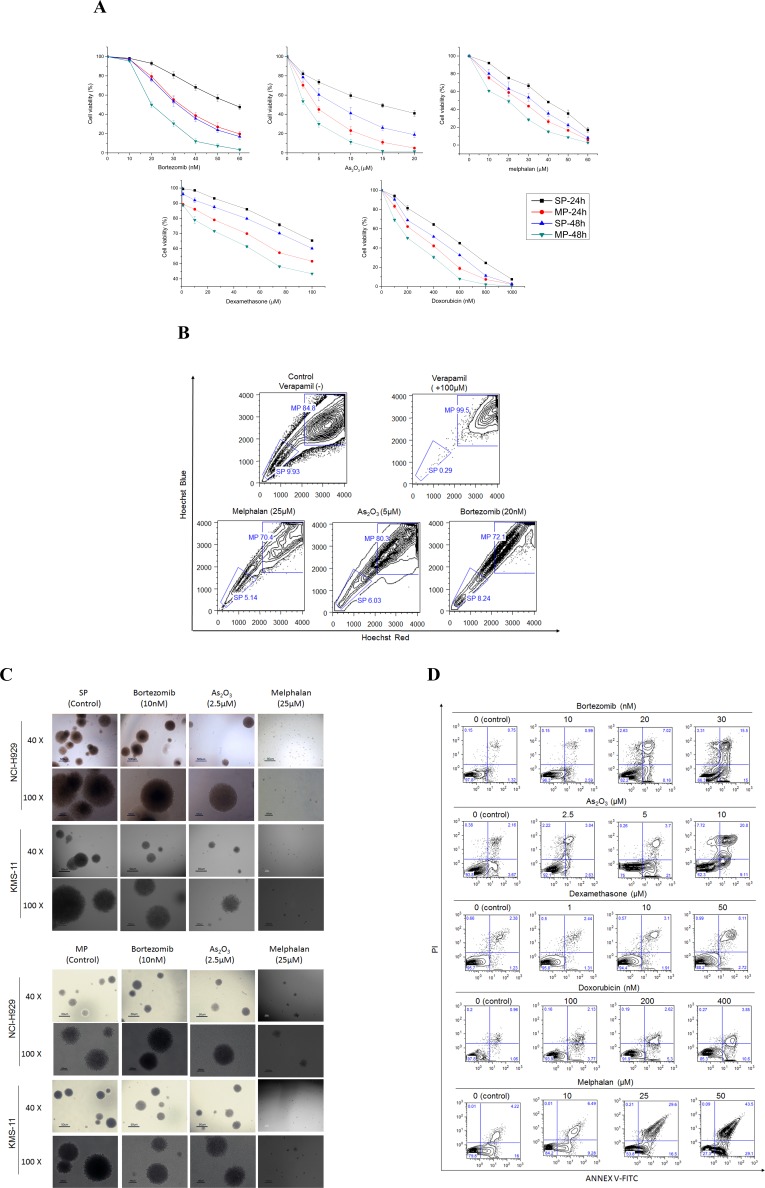
SP cells are resistant to chemotherapy agents **A**. Classical or novel clinically active agents: bortezomib, As_2_O_3_, dexamethasone, melphalan, or doxorubicin mediate NCI-H929 SP cell viability for 24 and 48 hours as measured by CCK-8 assay. **B**. Percentage of SP and MP fraction in KMS-11 cells after treating with melphalan, As_2_O_3_, and bortezomib for 48 hours. Cells disappeared with verapamil treated at 100μM as control. **C**. CFC assay showed the effect of bortezomib, As_2_O_3_, melphalan on SP and MP cells from NCI-H929 and KMS-11 cell lines. (200 cells/plate, magnification 40 ×, 100 ×). **D**. SP cells from NCI-H929 cell line were treated with bortezomib, As_2_O_3_, dexamethasone, melphalan, or doxorubicin for 48 hours, and apoptosis was measured with Annexin V/PI binding assay by flow cytometry. A representative graph from 3 independent experiments is shown (*p* < 0.05; n=3).

To determine whether classical or novel clinically active agents mediate SP cell viability, SP or MP cells were treated with anti-myeloma drugs bortezomib, As_2_O_3_, dexamethasone, melphalan, or doxorubicin with increasing concentrations for 24 and 48 hours. Cell viability was then measured by CCK8 assays. As shown in Fig. 2A, active agents significantly decreased the viability of SP and MP cells in a time- and dose-dependent manner. We observed at least two-fold a half-maximal inhibitory concentration (IC50) concentration in inhibitory rates of SP cells compared to MP cells in all test drugs. This may be one of the obvious triggers for relapse, since conventional treatment fails to affect the active stem-like MM cells.

To determine the effects of these agents on reducing the number of SP cells, we assessed the SP and MP fraction after 48 hour treatment with the various drugs. We found that As_2_O_3_ and melphalan could significantly decrease the percentage of SP cells. However, in contrast to Nara's study, which may be due to the different myeloma cell type, bortezomib only slightly reduced SP fraction even in relatively high concentrations (Fig. 2B) [8]. Dexamethasone and doxorubicin did not affect SP cells. All these drugs decreased MP fraction compared to control.

We next evaluated the effect of the drugs on growth of SP cells. Similar results from NCI-H929 and KMS-11 SP and MP cells were obtained. Approximately 50% decrease in colony number and formation were observed in presence of bortezomib and As_2_O_3_, while nearly no colony formation in cells treated with melphalan (Fig. 2C). These results indicate that these drugs hampered growth of potential SP cells, whereas SP and MP cells was unchanged by treatment with dexamethasone or doxorubicin.

Subsequently, we evaluated the effects of the drugs on the SP cell cycle. None of the drugs altered the cell cycle distribution in SP cells compared to controls (data not shown). Apoptosis by using annexin V-binding assay in SP cells treated by bortezomib, As_2_O_3_, or melphalan increased dramatically in a time- and dose-dependent manner, whereas dexamethasone or doxorubicin treatment failed to induce apoptosis in SP cells (Fig. 2D). Therefore, we speculated that the ability of bortezomib, As_2_O_3_, or melphalan to decrease clonogenicity to confer drug resistance in SP cells was derived from apoptosis.

### SP cells showed the potential distinct miRNA expression pattern

**Figure 3:**
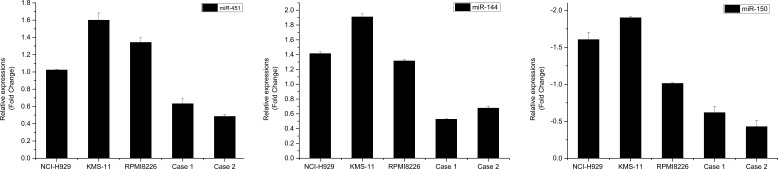
qRT-PCR validation of miRNA expression in SP cells The column indicates the relative fold change compared to MP cells. Each sample was analyzed in triplicate and was normalized to U6. Fold change was calculated by 2^−ΔΔCt^ method. The results were consistent with the microarray data.

We performed MiRCURY™ LNA array analysis of sorted SP and MP cells from two relapsed myeloma patients with more than 70% bone marrow plasma cells from bone marrow smear. Among the miRNAs exhibiting at least two-fold and statistically significant difference (*p* < 0.05) in expression, 43 miRNAs were identified in total. Ten of which were upregulated and 33 were downregulated in SP cells compared with those in MP cells (Table 1). Of these potential distinct miRNA expression pattern, miR-451, miR-144 and miR-150, were of interest because they have been reported to be involved in pathological mechanism of CSCs [20, 21]. The expression of these three miRNAs were further confirmed by qRT-PCR based on 2^−ΔΔCt^ methods (Fig. 3).

### Differential expression of miRNAs regulated PI3K/Akt/mTOR signaling pathways in SP cells

It is well known that genes do not work in isolation. Instead, complex molecular networks and cellular pathways are often involved in disease pathogenesis [22, 23]. We therefore performed pathway analysis of the predicted targets using KEGG to define the biological networks affected by differentially expressed miRNAs and their targets in SP cells. The list of the top pathways highly related to SP cells ranked by the enrichment *p* < 0.005 is presented in Supplemental Table 1 (FDR < 0.05). Out of all these pathways, which have been successively reported to play a determinant role in CSCs, PI3K/Akt/mTOR signaling appears to act as a crucial pathway in disease progression and development of therapeutic resistance [24, 25]. However, the role of PI3K/Akt/mTOR signaling in the maintenance of myeloma CSCs has not been clarified. We next confirmed the fidelity and reliability of the representative target genes located in PI3K/Akt/mTOR pathway by western blot analysis. Our results showed PI3K/Akt/mTOR proteins, p-S6 and p-P70S6K, to be significantly upregulated in SP cells compared with those of MP cells (Supplemental Fig. 2). Therefore, we speculated that PI3K/Akt/mTOR pathway may be regulated by specific miRNAs to exert its biological effects in MM SP cells.

### miR-451 enhanced the potentiated drug synergism effect in SP cell

To evaluate whether any of the 43 aberrantly expressed miRNAs previously identified in SP cells could target the core regulators in PI3K/Akt/mTOR, we used computational methods to predict the *TSC1* and *CAB39* which might be targeted by miR-451, expression in this pathway. Notably, miR-451 has been previously confirmed to be upregulated in SP cells (Fig. 3) and shown to maintain CSC properties in other diseases [21]. To examine the effects of miR-451 in MM SP cells, qRT-PCR confirmed a remarkable downregulation of miR-451 expression in SP cells after transfection of the mature sequence of miR-451 inhibitor (miR-451-inh) at 100nM (Supplemental Fig. 3).

**Figure 4:**
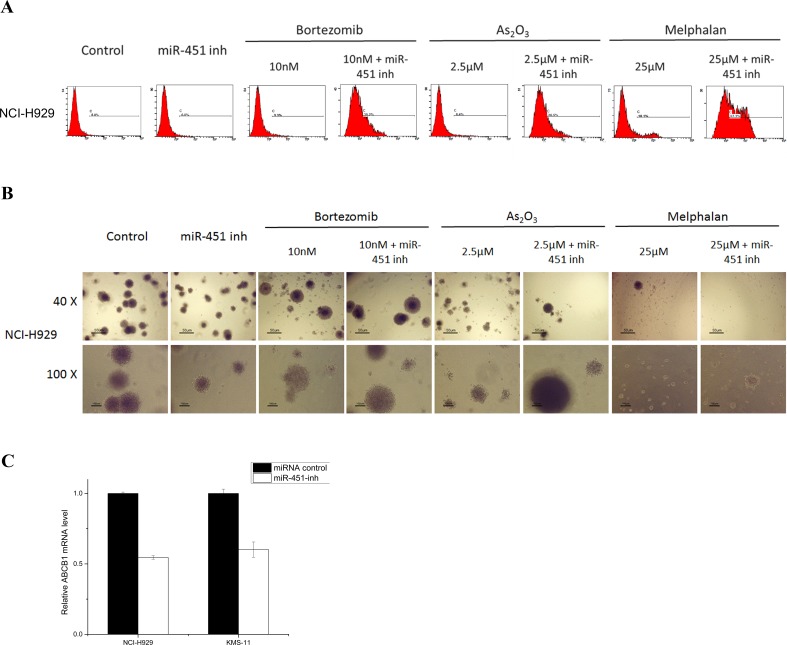
The potentiated drug synergistic effect of reducing miR-451 expression on SP cells **A**. Cell apoptosis was detected in SP cells from NCI-H929 cell line transfected with miR-451-inh treated with bortezomib, As_2_O_3_, and melphalan by flow cytometry. **B**. The clonogenic capacity was determined in SP cells from NCI-H929 cell line transfected with miR-451-inh treated with bortezomib, As_2_O_3_, and melphalan (400 cells/plate, magnification 40× and 100×). **C**. qRT-PCR showing relative MDR1 mRNA levels in NCI-H929 and KMS-11 SP cells transfected with miR-451-inh compared to control.

Unexpectedly there was no difference in cell proliferation, apoptosis, cell cycle status, or clonogenicity between SP and MP cells from NCI-H929 and KMS-11 at any time point after transfection of miR-451-inh oligonucleotides (data not shown). Intriguingly, miR-451 might have a potential anti-myeloma SP cell synergistic effect against CSC characteristics with bortezomib, As_2_O_3_, and melphalan treatments when SP cells were transduced with miR-451 inhibitor. Similar results were observed from NCI-H929 and KMS-11 cell lines for after 48 hour treatment. Representative NCI-H929 SP cells results are shown in Fig. 4. In apoptosis assay, SP cells transfected with miR-451-inh showed a significant increase in apoptosis compared to treatment with each agent alone, indicating that the reduced levels of miR-451 enhanced the efficiency of bortezomib, As_2_O_3_, or melphalan on SP cells (Fig. 4A, *p* < 0.05). Similarly, combination of miR-451-inh with anti-myeloma drugs significantly decreased colony formation compared to treatment with each agent alone, indicating that the low levels of miR-451 restrained SP cell proliferation (Fig. 4B, *p* < 0.05).

One of the underlying mechanisms for such drug synergism effect might be associated with ABC transporters. As shown in Fig. 4C, qRT-PCR data shows a near 50% decrease of ABCB1 in sorted SP cells after treatment with miR-451-inh compared to controls. Only slight changes were detected in ABCG2, ABCC2 and ABCC4 mRNA expression of SP cells (Supplemental Fig. 4). These results suggests that miR-451 enhanced the drug synergistic effect in SP cells might contribute to affect the ABC transporter pump function by regulating its family, especially in ABCB1, but further function research is needed to confirm this result.

### miR-451 targeted TSC1 gene to regulate PIK3/Akt/mTOR signaling in SP cells

**Figure 5:**
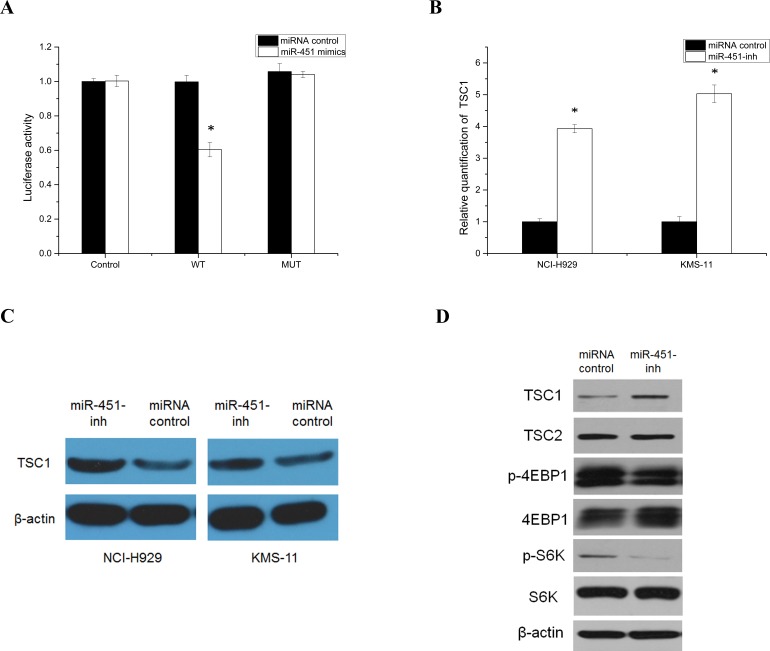
Identification and analysis of *TSC1* as a target gene of the miR-451 **A**. Direct targeting of *TSC1* 3′UTR by miR-451. NCI-H929 cells were co-transfected with luciferase/*TSC1* 3′UTR reporter vector (WT) and miRNA negative control or miR-451-mimics. A reporter vector with a mutated miR-451-binding site in the *TSC1* 3′UTR (MUT) was used as a control. Luciferase levels are expressed as mean relative to controls ± SD; *p < 0.05. Relative quantitation of *TSC1*, using qRT-PCR B. and western blot analysis **C.** in NCI-H929 SP cells following transfection with the miR-451 inhibitor (*p < 0.05). **D**. Inhibition miR-451 expression to further block PIK3/AKT/mTOR signaling activity in NCI-H929 SP cells.

The putative corresponding target *TSC1* was of particular interest due to its known function in a wide array of cellular processes through negative control of mTORC1 involving the PIK3/Akt/mTOR regulation. To evaluate whether abnormally expressed miR-451 could target *TSC1*, which has a highly conserved binding site in the 3′UTR of *TSC1* predicted by miRanda, and TargetScan, a luciferase reporter assay was used to study. The activity of *TSC1* luciferase was blocked by overexpression of miR-451 compared with control. This was abrogated by mutation at the miR-451 binding site, demonstrating a direct effect of miR-451 on the 3′UTR of *TSC1* (Fig. 5A). Furthermore, inhibition of miR-451 led to an increase in transcription and translation of *TSC1* in SP cells (Fig. 5B and 5C). Moreover, inhibition of miR-451 reduced downstream effectors, phospho-S6 and phospho-4EBP1, by inhibiting PIK3/AKT/mTOR signaling pathway in SP cells (Fig. 5D).

### A novel mTOR inhibitor agent S14161 targets MM SP cells specificity

**Figure 6:**
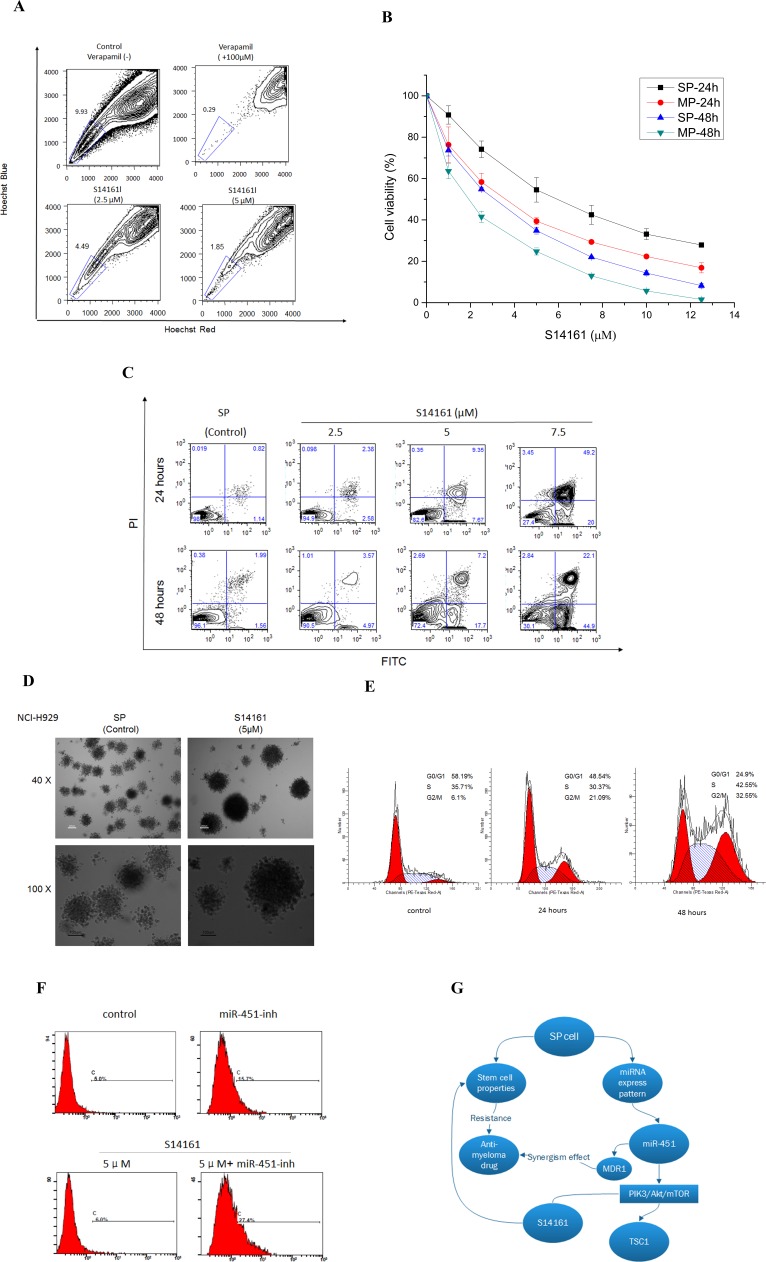
A novel mTOR inhibitor agent S14161 regulates SP cells stem cell properties **A**. S14161 decreased the percentage of SP fraction in NCI-H929 cells with a dose-dependent manner at 72 hours and cells treated with verapamil (100μM) as the negative control. **B**. S14161 mediated NCI-H929 SP cell validity for 24 and 48 hours measured by CCK-8 assay. **C**. SP cells from NCI-H929 cell were treated with S14161 for 24 or 48 hours, and apoptosis was measured with Annexin V/PI binding assay by flow cytometry. **D**. CFC assay shows the effect of S14161 on SP cells from NCI-H929 cell line. (600 cells/plate, magnification 40 ×, 100 ×). **E**. S14161 induced SP cell cycle from G0 to G2/M phase in a time-dependent manner. **F**. SP cells treated with S14161 and inhibiting mir-451 expression prompt apoptosis by flow cytometry. Representative graph from 3 independent experiments is shown (*p* < 0.05; n=3). **G**. The model of this study hypothesis shows potential mechanisms by which miRNA regulates biologic relevance in MM SP cells through PIK3/Akt/mTOR signaling.

From the results so far, it is clear that the PIK3/Akt/mTOR pathway may be important for maintaining SP cell features. To confirm this, we examined the effect of inhibiting PIK3/Akt/mTOR pathway on SP cells. Rapamycin, a specific classical inhibitor of mTOR, was firstly tested for its ability to reduce the SP fraction in NCI-H929 cells. Treatment with rapamycin at 5μM for three days, resulted in a significant decrease in SP fraction, from 1.54% to 0.28% (Supplemental Fig. 5). Recently, a small chemical compound, S14161, was identified as a PI3K inhibitor. S14161 displayed no gross toxicity at concentrations up to 500 mg/kg *in vivo*, making it a good candidate for use in myeloma and leukemia treatment in preclinical activity [26]. Thus, we were in particular interested in testing S14161 against SP cells. As shown in Fig. 6A, S14161 drastically reduced the SP fraction within NCI-H929 cells in a dose-dependent manner after 72-hour treatment. The ability of the S14161 to decrease the SP fraction was at least partially due to its ability to preferentially inhibit the SP cells. To support this, we treated NCI-H929 SP and MP cells with S14161 (0~12.5μM) for 24 and 48 hours and evaluated MM cell viability using CCK-8 assay. The SP cells treated for 24 hours had IC50 < 7.0μM, which were higher than MP cell lines (Fig. 6B). We further observed that SP cells after treatment with 2.5~7.5μM S14161 for 24 or 48 hours, showed increased apoptosis rate in a time- and dose-dependent manner (Fig. 6C). In addition, S14161 dramatically reduced the colony forming capacity of SP cells in comparison to control (Fig. 6D). Because S14161 decreased levels of D-cyclins, we tested the effects of S14161 on cell-cycle progression. In contrast to previous report [26] and MP cells data from our study (not shown), we measured that S14161 at 5μM induced SP cell cycle arrest at G2/M phase, with a corresponding reduction in the number of G0 quiescent cells in a time-dependent manner (Fig. 6E), which might be mediated others mechanism to effect cell cycle on SP cells. Furthermore, S14161 exhibited synergistic apoptotic effect when administered in conjunction with miR-451 (Fig. 6F). Taken together, the effect of the PI3K inhibitor S14161 in SP cells and the synergistic activity with the miR-451 inhibitor support the notion that S14161 may have potent activity not only in myeloma cells also in MM SP cells.

## DISCUSSION

Although there are several viewpoints on the stem-like properties within MM cells, the definitive MM CSCs phenotype/markers are still under investigation [27]. In this study, we used the efflux of Hoechst dye to identify the SP phenotype and isolate MM cancer stem-like cells.

We first identified SP cells which remained consistently a small between various experiments and over a panel of myeloma cells. As expected, no distinctive phenotypic features were identified between SP and MP cells. Consistent with previous studies [5, 10, 13], we observed CSC properties (i.e. cell cycle status, clonogenicity, and high drug efflux capacity) in SP cells. In cell cycle analysis, SP cells contain larger percentage of quiescent population than MP cells, which may be one of the reasons for the SP-induced drug resistance. Furthermore, we confirmed that SP cells contain clonogenic cells with high proliferative ability, a distinguished property of CSCs for producing less-differentiated progeny and undergoing self-renewal [5], resulting in generation of a significant proportion of cells in comparison with MP cells. Although the proliferation rates were low compared to others reports [8, 13], it might be due to the low amount of SP cells used per plate for colony formation assay. Moreover, we still observed a significantly higher tumorigenicity in SP cells compared to MP cells through our *in vitro* experiments and *in vivo* study of SP cell tumorigenicity, though with limited data due to the rare numbers of SP cells from FACS. Combined with previous studies [5, 13], we have generated a solid foundation in confirming SP cells as a source of tumorigenicity of myeloma for CSC studies.

Many studies have demonstrated CSCs as an important cause of drug resistance and relapse due to their regulation of ABC activity in drug efflux [5, 10, 13]. Similarly, we observed an elevated expression of ABCG2 in SP cells, confirming ABCG2 as a molecular marker of SP phenotype [18]. We also observed high levels of ALDH activity in SP cells, which has been previously shown to also be high in myeloma stem-like cells [5, 28]. However, ALDH was not an eligible marker for myeloma CSCs in our study since over 50% of SP cells fail to meet the criteria for being defined as CSCs.

The CSC hypothesis states that a small subset of cells with in a cancer population is intrinsically more resistant to therapy than the rest of the tumor cells [6]. Previous studies have reported CD138-/CD19+/CD20+/CD27+ MM stem cells to be resistant to dexamethasone, lenalidomide, cyclophosphamide and bortezomib [5]. However, in other studies, MM SP cells were susceptible to lenalidomide [13] or bortezomib treatment [8]. Here we expanded the drug resistance analysis to a panel of active clinical agents and showed that SP cells were more resistant to bortezomib, As_2_O_3_, dexamethasone, melphalan, and doxorubicin compared to MP cells. Notably, we show SP cells to be susceptible to bortezomib, As_2_O_3_, and melphalan treatment as seen from the decreased SP fraction, significantly increased SP cells apoptosis, as well as the lowered percentage of SP population and clonogenicity at clinically achievable concentrations.

To the best of our knowledge, this is the first study to characterize and compare miRNA signatures of MM SP cells with MP cells. Of the abnormally regulated miRNAs, we looked at the molecular mechanisms involved in miR-451-mediated effects. Cells expressing miR-451 has been demonstrated to exhibit CSCs properties in colon carcinoma [21, 29]. Although miR-451 has yet been proven directly effective against myeloma SP cells in proliferation, apoptosis, or self-renewal, we found miR-451 to potentiate the effects of bortizomib, As_2_O_3_, and melphalan through enhancing apoptosis and inhibit clonal sphere formation in SP cells.

We further found that treatment with miR-451 inhibitor decreased the expression of MDR1 mRNA expression in MM SP cells, which may confer its synergistic effects. Previous study reported that miR-451 increased MDR1 expression in parental cell line A2780 [30]. Taken together, our results suggests that the effects of miR-451 on MDR1 expression appear to modulate anti-myeloma agents' effectiveness by inhibiting the expression of certain transcriptional factors involved in suppressing *MDR1* gene activation. The precise mechanisms require further investigation.

Until now, several pathways, such as the hedgehog [10] and Notch pathways [6], have been shown to be involved with maintaining myeloma stem cell properties. The role of PI3K/Akt/mTOR signaling in the maintenance of CSCs has been addressed in other tumors, but the conclusions of these reports are controversial [25, 31]. We identified the effects of miR-451 on the activation of the PI3K/Akt/mTOR pathway and its downstream targets to stimulate stemness features through direct targeting of *TSC1*. Disruption of PI3K/Akt/mTOR signaling by constitutive miR-451 expression alters the activity of pathways downstream of *TSC1*, including reduced S6 and 4EBP1 phosphorylation. Thus, miR-451 targets *TSC1* to mediate PI3K/Akt/mTOR signaling, which plays an essential role in myeloma stem cell biology.

A novel specific inhibitor, S14161, inhibits D-cyclin transactivation via the PI3K signaling pathway to exert anti-myeloma activity [26], while displaying no gross toxicity at concentrations up to 500 mg/kg in an *in vivo* assay, making it a probable drug candidate. More importantly for myeloma SP cells, S14161 exerted multiple actions including directly decreasing SP fraction, inhibiting SP proliferation, increasing apoptosis, inducing cell cycle progression, as well as synergizing with low miR-451 expression. Taken together, these suggest a significant potential target for myeloma stem and cancer cell therapeutics. The overexpression of D-cyclins contributes to the pathogenesis and chemoresistance of MM [32]. Our previous studies have demonstrated the ABCG2 activity of SP cells and the MDR1 mediated miR-451 effects. Collectively, we believe that S14161 may down-regulate D-cyclins via a MDR pathway mediated by ABCG2 or MDR1, which controls or bypasses these events to affect SP features.

In summary, our results demonstrate that MM SP cells exhibit stemness, and the PI3K/Akt/mTOR pathway is activated in SP cells. Regulating *TSC1* expression by miR-451 and related downstream targets (Fig. 6G) may be relevant to the pathophysiology of the disease, and miR-451 may serve as a potential therapeutic target.

## MATERIALS AND METHODS

### Culture of MM cell lines and primary MM cells

A panel of myeloma cell lines NCI-H929, RPMI 8226, KMS-11, LP-1, U266, SKO, and primary myeloma cells were used, which were cultured as described in Supplemental Methods.

### Drug, reagents and antibodies

Bortezomib was kindly provided by Xian-Janssen Pharmaceutical Ltd. Rapamycin (Sirolimus) was purchased from Selleck Chemicals LLC (Houston, TX, USA). 8-ethoxy-2-(4-fluorophenyl)-3-nitro-2H-chromene (S14161) was kind provided by Dr. Mao (Cyrus Tang Hematology Center, Soochow University, Suzhou, Jiangsu, China) [26]. Other chemicals were purchased from Sigma Company (Saint Louis, MO), unless specifically annotated. PI3K/Akt/mTOR pathway related primary antibodies were purchased from Abcam (Cambridge, MA, USA), and others primary antibodies were purchased from Cell Signaling Technology (Danvers, MA, USA).

### Flow cytometric analysis

SP sorting analysis was performed with Hoechst 33342 dye method as described by Goodell et al. [9] with modifications. Aldehyde dehydrogenase (ALDH) activity was tested by the Aldefluor reagent (Stem Cell Technologies, Vancouver, Canada). Cell apoptosis assay was detected by using annexin V-binding assay, and immunophenotyping study was performed with mouse anti-human related antibodies. These methods were described in detail in Supplemental Methods.

### MiRNA profiling and analysis

To screen the miRNAs profiling between SP and MP cells, miRCURY™ LNA Array (v.16.0, Exiqon) was used according to the manufacturer's instructions, as described in the Supplemental Methods. The microarray data were deposited on the Gene Expression Omnibus (accession number GSE56163).

### Bioinformatic analysis based on miRNA expression profile

MiRNA target was predicted by miRanda, and TargetScan, as well as the miRNA analysis involved generation of pathway networks using the Kyoto Encyclopedia of Genes and Genomes (KEGG), as described in Supplemental Methods.

### Quantitative real-time PCR assay (qRT-PCR)

qRT-PCR was performed using the quantitative SYBR Green PCR kit following the manufacturer's protocol, as described in Supplemental Methods. All quantitative the fold change was calculated by the 2^−ΔΔCt^ method.

### RNA oligonucleotide and cell transfection

MiRNA mimics, miRNA inhibitors, and their cognate control RNAs were purchased from Ambion (Austin, TX, USA) or Genepharma (Shanghai, China). Transfection was performed using SuperFection (Pufei, USA) transfection reagent according to the manufacturer's instructions. Transfection efficiency (>90%) was confirmed with the use of the Silencer 6-carboxy-fluo-rescine (FAM)-labeled Negative Control.

### Cell-based assays

Cell viability was tested by colorimetric assay kit (Dojindo Laboratories, Tokyo, Japan) based on the MTT assay. Clonogenicity activity was detected by colony-forming cell (CFC) assay. Both methods were described in detail in Supplemental Methods.

### Western blot analysis

Cell lysates and total protein concentration were measured with the BCA Protein Assay Kit (Pierce Biotechnology, Rockford IL, USA). The proteins of interest were detected, as described in Supplemental Methods.

### Luciferase assay

The luciferase activity was measured using the Dual-Luciferase Reporter Assay System (Promega), as described in Supplemental Methods.

### Animal models

SP or MP cells from NCI-H929 cell lines were injected subcutaneously together with Matrigel basement membrane matrix (Becton Dickinson) into NOD/SCID mice approved in accordance with the institutional guidelines for the use of laboratory animals; details were described in Supplemental Methods.

### Statistical analysis

Data were expressed as the mean plus or minus SD. Analysis of statistical significance between groups was made using a 2-tailed unpaired Student's t test. A value of *p* < 0.05 was considered statistically significant. All experiments were performed in triplicate with consistent results.

## SUPPLEMENTARY MATERIALS, FIGURES, TABLES


