# Supplementary material for: MicroRNA-451 regulates stemness of side population cells via PI3K/Akt/mTOR signaling pathway in multiple myeloma
Source: Oncotarget. 2015 Apr 12;6(17):14993–5007. doi: 10.18632/oncotarget.3802 (PMC4558131; doi:10.18632/oncotarget.3802)
Supplement: Supplementary file 1 [file oncotarget-06-14993-s001.pdf]

MicroRNA-451 regulates stemness of side population cells via PI3K/Akt/mTOR signaling pathway in multiple myeloma

Supplementary Material

Supplemental Methods:

Culture of MM cell lines and primary MM cells

Myeloma cell lines NCI-H929, RPMI 8226, KMS-11, LP-1, U266, and SKO were cultured in RPMI 1640 medium (GIBCO-BRL, Grand Island, NY) supplemented with 10% heat-inactivated fetal bovine serum (FBS), 100 U/mL penicillin, 100 µg/mL streptomycin, and 2 mmol/L l-glutamine (GIBCO-BRL) at 37°C in humidified air containing 5% carbon dioxide. Myeloma cell line NCI-H929 was a gift from Dr. Margaret H.L. Ng (Prince of Wales Hospital, Chinese University of Hong Kong), and KMS-11 was a generous gift from Dr. Xinliang Mao (Cyrus Tang Hematology Center, Soochow University, Suzhou, Jiangsu, China), RPMI 8226, LP-1, U266 as well as SKO cell lines were obtained from the American Type Culture Collection (ATCC; Manassas, VA, USA). Culture medium was replaced every 2 days. All experiments were conducted with cells in logarithmic phase.

Primary myeloma cells were isolated from the bone marrow samples of seven MM patients receiving routine diagnostic aspiration, with informed consent approved by the Institutional Review Board at Changzheng Hospital. All participants provided written informed consent in accordance with the Declaration of Helsinki. The fresh cells were cultured overnight to pre-activate in RPMI 1640 medium (GIBCO-BRL, Grand Island, NY, USA), supplemented with 10% FBS, 100 U/mL penicillin, 100 µg/mL streptomycin, and 2 mmol/L l-glutamine during ex vivo culture, and maintained at 37°C in 5% carbon dioxide.

Drug, reagents and antibodies

For drug resistance studies, SP and MP were isolated individually from the NCI-H929 or KMS-11 cell line, the cells were cultured in RPMI 1640 containing 10% fetal bovine serum alone or with multiple drugs, including bortezomib (10nmol/L; 20nmol/L; 30nmol/L), As_2O_3 (2.5µm; 5µm; 10µm), dexamethasone (1µm; 10µm; 50µm), melphalan (10µm; 25µm; 50µm), doxorubicin (100nm; 200nm; 400nm), or S14161 (2.5µm; 5µm; 7.5µm) for 24 hours, 48 hours, or 72 hours, respectively. Bortezomib was kindly provided by Xian-Janssen Pharmaceutical Ltd. Rapamycin (Sirolimus) was purchased from Selleck Chemicals LLC (Houston, TX, USA). 8-ethoxy-2-(4-fluorophenyl)-3-nitro-2H-chromene (S14161) was kind provided by Dr. Mao (Cyrus Tang Hematology Center, Soochow University, Suzhou, Jiangsu, China).[18] Other chemicals were purchased from Sigma Company (Saint Louis, MO), unless specifically annotated. mTOR pathway related primary antibodies were purchased from from Abcam (Cambridge, MA, USA), and others primary antibodies were purchased from Cell Signaling Technology (Danvers, MA, USA).

Flow cytometric analysis

For Hoechst 33342 staining and SP cells sorting: cells were washed with PBS and resuspended in above mentioned culture RPMI medium at $1X10^6$ /mL. The cells were incubated with Hoechst 33342 (Sigma, St. Louis, MO) at 5µg/ml either alone or together with ABC transporter inhibitors verapamil (100µM, Sigma), or reserpine (50 mmol/L, Sigma), for 120 minutes at 37°C. After staining, the cells were centrifuged and resuspended in ice-cold PBS containing 1µg /mL propidium iodide (PI) and maintained at 4°C for flow cytometry analysis and sorting. Cell analysis and sorting were determined by a MoFlo cytometer (Beckman Coulter, Inc.). Hoechst dye was excited at 407 nm by trigon violet laser, and dual wavelengths were detected by 450/40 (Hoechst 33342-Blue) and 695/40 (Hoechst 33342-Red) filters.

Aldehyde dehydrogenase (ALDH) activity was tested by the Aldefluor reagent (Stem Cell Technologies, Vancouver, Canada) according to manufacturer's instructions, and diethylaminobenzaldehyde (DEAB) was used as control.

For apoptosis assay: cell apoptosis was detected by using annexin V staining. MM cells were cultured in media alone, or with media plus with various concentration agents treatment in culture medium for 24 and 48 hours. Cells were then washed twice with ice-cold PBS and resuspended (1×10^6 cells/mL) in binding buffer (10mmol/L HEPES, pH7.4, 140mmol/L NaCl, 2.5mmol/L $CaCl_2$). MM cells (1×10^5) were incubated with annexin V-FITC (5µL; Pharmingen, San Diego, CA, USA) and propidium iodide (PI, 5mg/mL) for 15 minutes at room temperature. AnnexinV+PI- apoptotic cells were enumerated by using the flow cytometer (Beckman Coulter).

For immunophenotyping study: MM cell lines were stained with mouse anti-human CD138-PE, CD38-PECY5, CD19-ECD, and CD20-ECD antibodies and then analyzed with FC500 flow cytometer (Beckman Coulter, Fullerton, CA, USA)

MiRNA profiling and analysis

Total RNA was harvested using TRIzol (Invitrogen) and miRNeasy mini kit (QIAGEN) according to manufacturer's instructions. After having passed RNA quantity measurement using the NanoDrop 1000, the samples were labeled using the miRCURY™ Hy3™/Hy5™ Power labeling kit (Exiqon) and hybridized on the miRCURY™ LNA Array (v.16.0). After hybridization, scanning was performed with the Axon GenePix 4000B microarray scanner (Molecular Devices, Downingtown, PA, USA). GenePix pro V6.0 (Molecular Devices) was used to read the raw intensity of the image. Background correction was performed using the "normexp + offset = 50" method, which provides variance stabilization and guaranties no negative values. Filtering was performed to identify miRNA up- or down- regulated at least 2.0-fold with a p-value of < 0.05 in SP cells compared with MP cells. The analysis was carried out using the limma package from Bioconductor according to the method (Smyth GK, Stat Appl Genet Mol Biol 3, 2004). The microarray data are deposited on the Gene Expression Omnibus (accession number GSE56163).

Bioinformatic analysis based on miRNA expression profile

For miRNA target prediction: microRNAs predicted to target gene of the miRNAs exhibited differential expression were predicted by mirBase (http://microrna.sanger.ac.uk), miRanda (http://www.microrna.org) and TargetScan (http://www.targetscan.org).

For pathway analysis: The network of the critical miRNAs and their targets was established according to the miRNA degree. The miRNA analysis involved generation of pathway networks using the Kyoto Encyclopedia of Genes and Genomes (KEGG, http://www.genome.jp/kegg/). A two-sided Fisher's exact test and chi-square test were used to classify the enrichment of pathway category, and the false discovery rate (FDR) was calculated to correct the p-value. The pathway had a p-value of < 0.005 and an FDR of < 0.05, which was chosen for further analysis. The regulator pathway annotation was performed on the basis of scoring and visualization of the pathways collected in the KEGG database as well.

Quantitative RT- PCR assay (Q-RT-PCR)

All RNA samples, including small RNAs, were purified by miRNeasy Mini Kit (QIAGEN), and then cDNAs were synthesized using a reverse transcription kit (TaKaRa Bio Inc, Japan). After reverse transcription, qRT-PCR was performed using the quantitative SYBR Green PCR kit with gene-specific primers (TaKaRa Bio Inc, Japan) following the manufacturer's protocol. Real-time quantitative PCR studies used the LightCycler® 480 instrument (Roche Diagnostics). All quantitative the fold change was calculated by the 2-△△ Ct method. All experiments were done in triplicates. The U6 snRNA was used as a control to normalize miRNA quantitative, while GAPDH was used as a control to normalize mRNA quantitative in the Q-RT-PCR assay.

RNA oligonucleotide and cell transfection

MiRNA mimics, miRNA inhibitors, and their cognate control RNAs were purchased from Ambion (Austin, TX, USA) or Genepharma (Shanghai, China). Transfection was performed using SuperFection (Pufei, USA) transfection reagent according to the manufacturer's instructions. Transfection efficiency (>90%) was confirmed with the use of the Silencer 6-carboxy-fluo-rescine (FAM)-labeled Negative Control. Total RNA and protein were collected for assay 48 or 72 hours after transfection.

Cell-based assays

Cell viability was tested by colorimetric assay kit (CCK-8 assay kit; Dojindo Laboratories, Tokyo, Japan) based on the MTT assay, according to the manufacturer's instructions. Briefly, $1\sim5\times10^3$ cells were incubated in 96-well plates with different concentration treatments in culture medium for different time points depend on experiment design, and then 10μL of the CCK-8 solution was added to each well. After 2 hours incubation at room temperature, the optical density (OD) was measured using a spectrophotometer (Molecular Devices Co., Sunnyvale, CA) and the fold-increase in the OD compared to that of the control (proliferation

index) was calculated.

Assays for colony-forming cell (CFC) activity were performed by plating the 4×10^2 or 2×10^3 sorted SP and MP cells in containing 1mL Iscove's MDM with 1% methylcellulose, 30% FBS, 10^{-4} M 2-mercaptoethanol medium (Methocult; Stem Cell Technologies, Vancouver, BC, Canada) supplemented with a cocktail of growth factors (50ng/mL rh stem cell factor, 10ng/mL rh GM-CSF, 10ng/mL rh IL-3, and 3U/mL erythropoietin) with or without drugs in duplicate cultures. The plates were incubated at 37°C in a humidified incubator with 5% CO_2 for 14 days, and the number of colonies was counted using an inverted microscope with 4 × and 10 × planar objective.

Western blot analysis

Cell lysates and total protein concentration was measured with the BCA Protein Assay Kit (Pierce Biotechnology, Rockford IL, USA). Equal amounts of protein were subjected to SDS-PAGE and proteins were transferred to nitrocellulose membranes (GE Healthcare, USA). The membrane was blocked in PBS containing 5% non-fat milk and 0.1% Tween-20, washed twice in PBS, and incubated with primary antibody at room temperature for 2 hours, followed by incubation with secondary antibody at room temperature for 45 minutes. Afterward, the proteins of interest were visualized using ECL chemiluminescence system (Santa Cruz Biotechnology, USA). ß-actin was used to normalize the amount of protein in each sample.

Luciferase assay

The hsa-miR-451 vector and pmirGLO, pmirGLO- tuberous sclerosis 1 (TSC1)-3'-UTR, or pmirGLO-TSC1 3'-UTR-mut were co-transfected into NCI-H929. To generate TSC1 3'UTR mutants containing mutations in the conserved miR-451 binding site, site-directed mutagenesis was performed using the wild-type 3'UTR as the template. In the 3'UTR mutant, the nucleotide sequence complementary to nt 2～5 of miR-451 was mutated to the same sequence as that in miR-451 (from CGG to TAA).Cell lysates were prepared at 24 hours post-transfection. The luciferase activity was measured using the Dual-Luciferase Reporter Assay System (Promega). The values obtained from hsa-miR-451 vector and pmirGLO were set as 100%.

Animal models

A total of 1×10^5 SP cells or MP cells from NCI-H929 cell lines were injected subcutaneously together with Matrigel basement membrane matrix (Becton Dickinson) into 7 or 8 weeks aged NOD/SCID mice, which were approved by the Second Military Medical University Institute Animal Care and Use Committee guidelines for the use of laboratory animals. Engraftment of SP or MP cells was monitored every 7 days and changes in tumor size were measured by calipers. The mice were sacrificed at day 100 in accordance with institutional guidelines. The tumors were surgically removed and pictures were performed using camera in Xenogen system, and then were immunostained with anti-CD138 and anti-CD38.

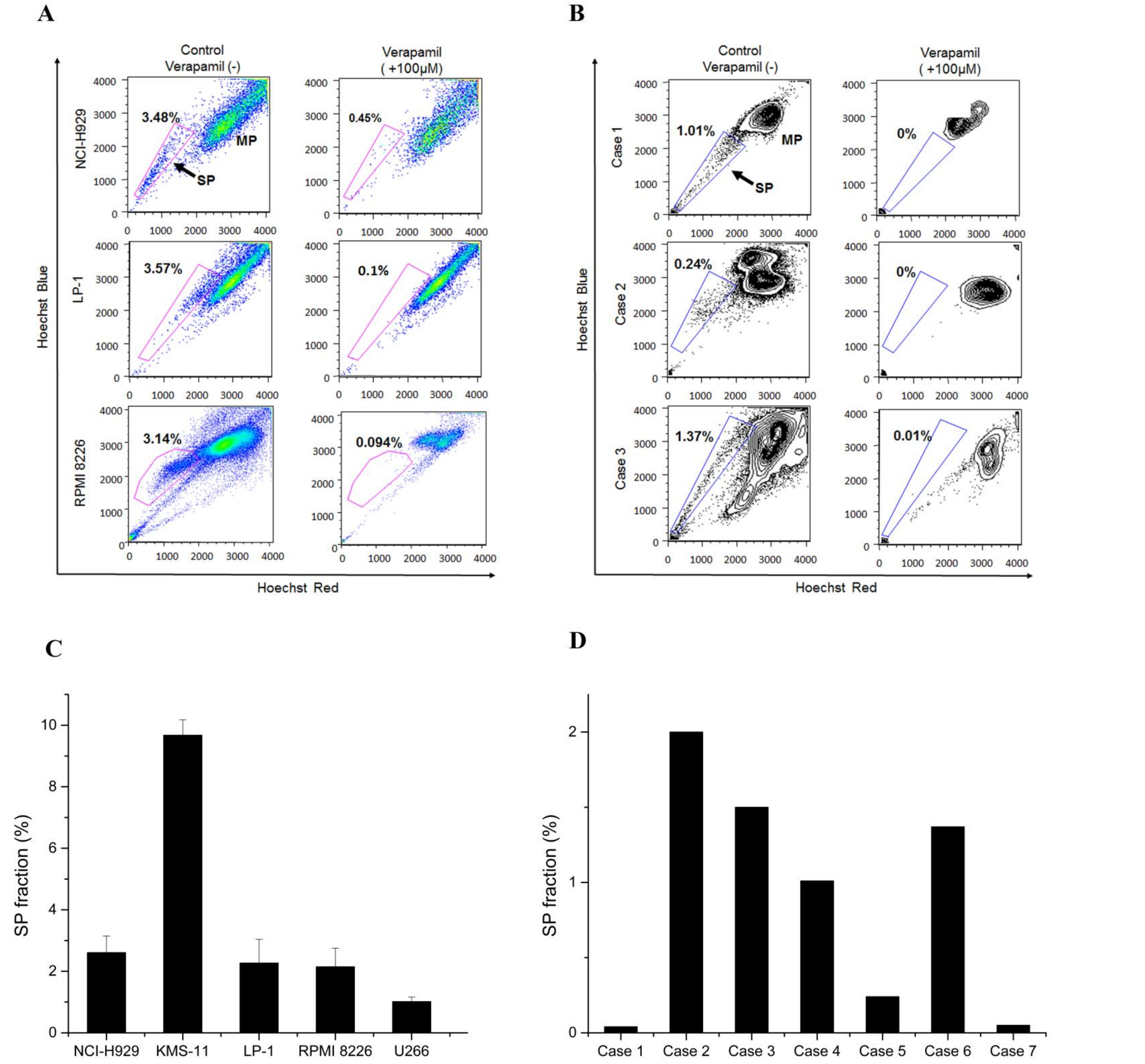

Figure S1: The percentage of SP cells varies from myeloma cell lines and primary cells. A. The flow cytometric dot plots present SP cells in NCI-H929, LP-1, RPMI 8226, and KMS-11 cells after incubating in Hoechst 33342 with or without 100μM verapamil as the negative control for SP cells. B. Representative flow diagram pictures showing of SP cells from the bone marrow of three representative MM patients. C. Percentage of SP fraction detected in a panel of MM cell lines. D. SP fraction were detected in seven MM patients' bone marrow samples. X-axis represents Hoechst red fluorescence intensity, while and Y-axis represents Hoechst blue fluorescence intensity. The gate represents the SP fraction in MM cells, which completely depleted by verapamil (100μM) in myeloma cell lines and primary cells.

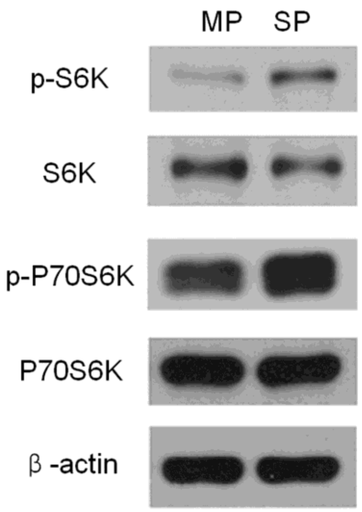

Figure S2: Western blotting validation differentially activated PI3K/Akt/mTOR signaling pathway in SP cells from KEGG pathway analysis.

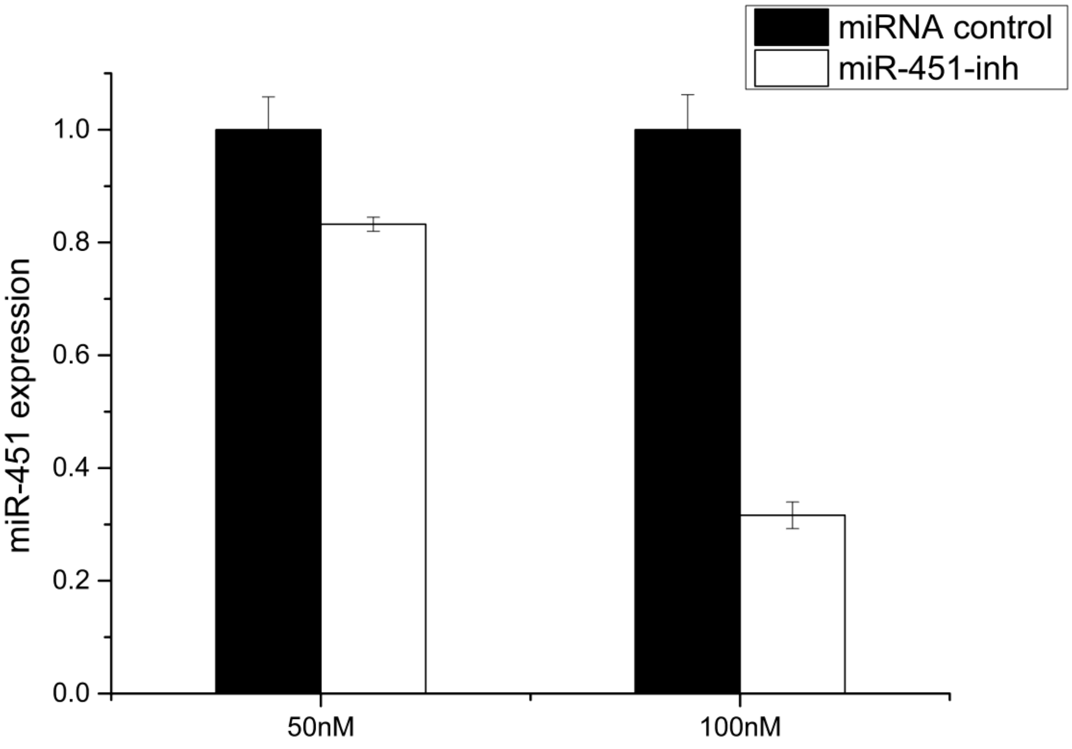

Figure S3: Down-regulation miR-451 expression by miR-451-inhibitor. SP cells were transfected with miR-451 inhibitor at different concentration (50nM or 100nM) and then cultured for 3 days. Q-RT-PCR showed that miR-451 expression was significantly inhibited at 100nM compared to the nontarget miRNA inhibitor transfected control cells.

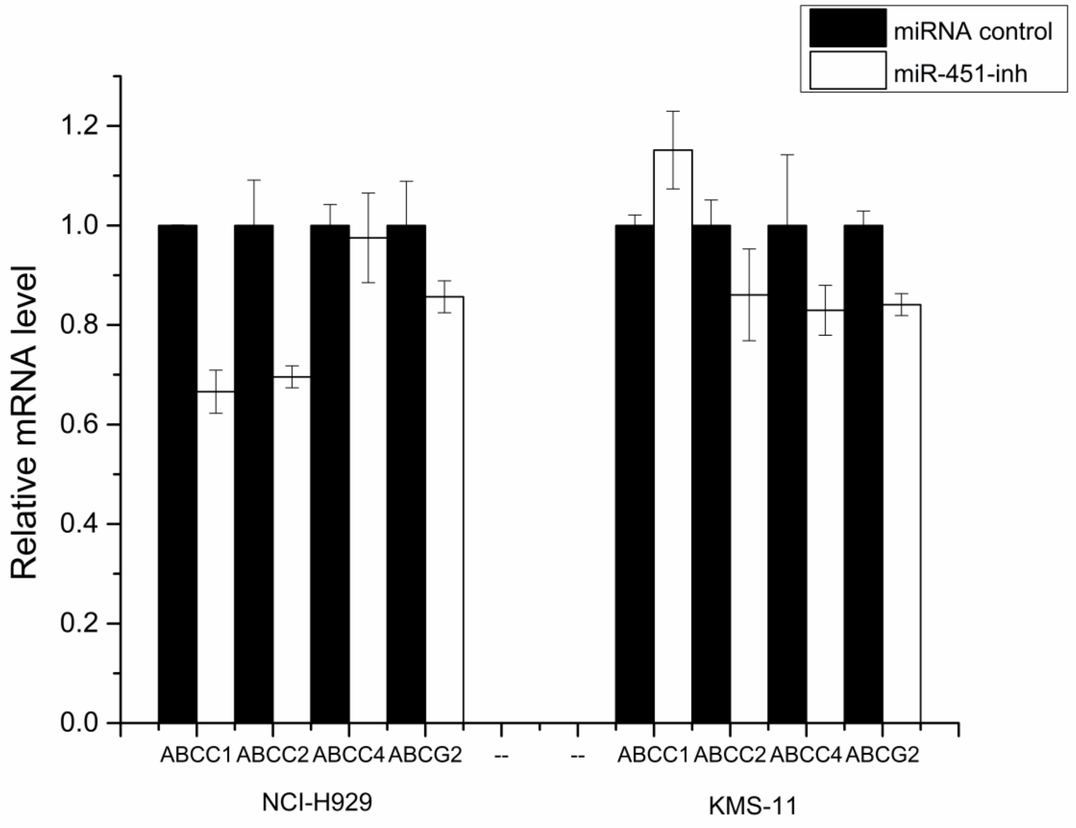

Figure S4: Q-RT-PCR showing relative ABC transporter including ABCC1, ABCC2, ABCC4, and ABCG2 mRNA levels in NCI-H929 and KMS-11 SP cells transfected with miR-451-inh compared to miRNA control.

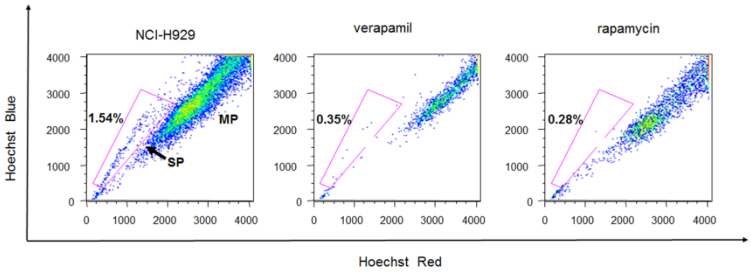

Figure S5: Rapamycin, a specific classical inhibitor of mTOR, decreased of the SP fraction within NCI-H929 cells. Cells treated with verapamil (100μM) as the negative control.

Supplements Table 1: Five highly significant (*p* < 0.005) biological pathways potentially affected by the differentially expressed miRNA in SP cells.

Pathway ID	Pathway	No. of Genes	Enrichment	FDR	*p*-value
hsa04010	MAPK signaling pathway	137	1.501649094	0.004636867	0.000143884
hsa04012	ErbB signaling pathway	57	1.924590282	0.004636867	0.000151673
hsa04310	Wnt signaling pathway	85	1.66460177	0.005564951	0.000208036
hsa04150	mTOR signaling pathway	37	2.090167381	0.011537165	0.000671005
hsa04350	TGF-beta signaling pathway	48	1.67859002	0.033097251	0.003711841
